# Outcomes of Growth Rod Distraction in Early Onset Scoliosis: A Case Series From a Tertiary Spine Centre

**DOI:** 10.7759/cureus.102361

**Published:** 2026-01-26

**Authors:** Sargunan B, Vishnu Prasath, Thomas John

**Affiliations:** 1 Spine Surgery, SKS Hospital and Post Graduate Medical Institute, Salem, IND

**Keywords:** case-series, distraction surgery, early-onset scoliosis, growth rod instrumentation, pediatric scoliosis

## Abstract

Early onset scoliosis (EOS) presents a unique challenge due to its potential to compromise thoracic growth and pulmonary development during childhood. Growth rod instrumentation offers a fusion-sparing approach that controls deformity while allowing spinal and thoracic expansion. This case series describes four children aged eight to 10 years with varying etiologies of EOS who underwent growth rod application followed by scheduled distraction procedures. Clinical records and radiographic findings were reviewed to document deformity magnitude, surgical intervention, serial lengthenings, and follow-up outcomes. All children showed progressive improvement in Cobb angle, thoracic height, and overall clinical alignment with each distraction, and none experienced neurological deficits, implant failure, or wound complications. Families also reported meaningful cosmetic and functional benefits. These observations support growth rod distraction as a safe and effective strategy for managing EOS in young children, promoting controlled deformity correction and thoracic development during critical growth years.

## Introduction

Early onset scoliosis (EOS) refers to spinal deformities that present before the age of ten and represent a unique clinical challenge due to their potential impact on thoracic growth and cardiopulmonary development. Progressive spinal curvature during this critical growth period can lead to thoracic insufficiency syndrome, impaired lung maturation, and long-term respiratory compromise if not effectively treated [1]. Traditional early spinal fusion, though capable of controlling deformity, restricts spinal and thoracic cage growth and is therefore associated with reduced pulmonary function and shortened trunk height [2]. To overcome these limitations, fusion-less surgical techniques such as traditional growing rods (TGR) have been developed to maintain deformity control while allowing continued spinal and thoracic expansion. Growing rod distraction at planned intervals has been shown to achieve meaningful curve correction, promote thoracic lengthening, and preserve pulmonary capacity during growth [3]. Growth rod distraction is a growth‑preserving surgical technique in which expandable rods are anchored to the spine above and below the deformity and periodically lengthened at planned intervals to accommodate spinal growth while maintaining curve control. Multiple studies have demonstrated that serial distractions can provide sustained deformity correction, though complications such as rod breakage, implant prominence, and the need for repeated surgeries remain important considerations [4]. The Cobb angle is a radiographic measurement used to quantify the severity of scoliosis, while thoracic height (measured from T1 to T12) reflects longitudinal thoracic and spinal growth. Given the importance of balancing deformity control with thoracic development, evaluating outcomes of growth rod instrumentation remains crucial. Each case was etiologically classified based on established clinical, radiographic, and syndromic features (including neurofibromatosis type 1 and congenital vertebral anomalies), with consistent documentation of presenting symptoms, neurological status, and curve characteristics to facilitate clinical identification despite etiological heterogeneity. This case series aimed to evaluate the radiological and clinical outcomes of traditional growing rod distraction in children with early onset scoliosis treated at a tertiary spine centre. This case series examines radiological and clinical outcomes following growth rod distraction in children with EOS.

## Case presentation

Case report one

**Figure 1:**
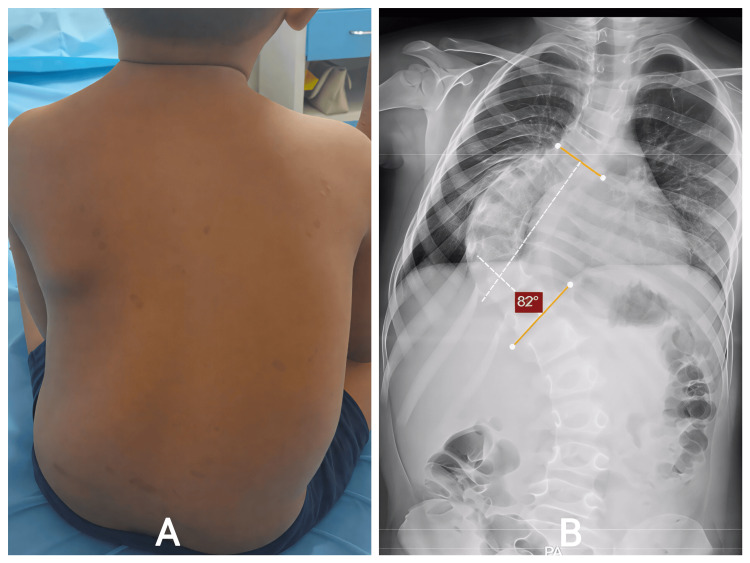
A: Posterior sitting view showing right thoracic prominence, truncal shift, and asymmetry consistent with dystrophic scoliosis in the child. B: Severe right thoracic scoliosis measuring 82.4° with vertebral rotation and dystrophic changes.

An eight-year-old male child with a known diagnosis of neurofibromatosis Type 1 was brought in with progressively worsening spinal deformity, first noted by his parents around the age of six. Initially, the deformity appeared mild, but over two years, they observed increasing truncal tilt, asymmetric shoulder height, and a prominent right thoracic rib hump. He denied back pain, limb weakness, gait disturbance, or bowel/bladder dysfunction. Examination revealed café-au-lait macules, thoracic asymmetry, right rib prominence, and mild compensatory lumbar shift. Neurological examination remained normal (Figure 1A). Standing full-length spinal radiographs showed a severe right thoracic curve of 82.4°, extending from the upper thoracic to the thoracolumbar junction, with associated kyphosis of 41°. Thoracic height (T1-T12) measured only 162 mm, reflecting restricted thoracic volume (Figure 1B). MRI excluded intraspinal anomalies. Given the aggressive curve, high progression risk, and his young age, early fusion was considered inappropriate because of the anticipated long-term cardiopulmonary compromise. Therefore, he underwent T3-L2 growth rod application with controlled deformity correction.

**Figure 2:**
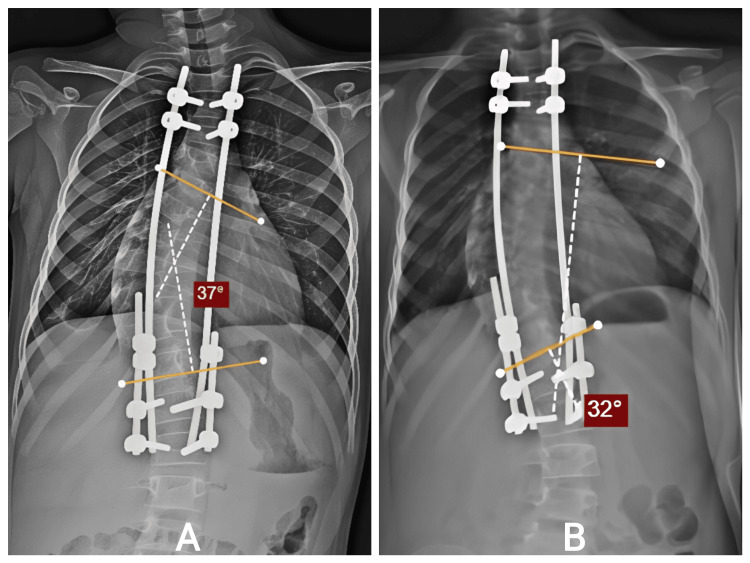
Follow-up Distraction Radiographs. A: After T3–L2 dual growth rods: Cobb reduced 82.4° → 37°, thoracic height 162 → 180 mm, kyphosis 41° → 37°. B: 32°, thoracic height 195 mm. T3: thoracic vertebra; L2: lumbar vertebra; mm: millimeters.

Intraoperatively, stable pedicle screw fixation was achieved proximally and distally. Gradual distraction allowed partial curve correction without compromising neurological function. Postoperative radiographs showed marked improvement: the Cobb angle decreased to 37°, thoracic height increased to 180 mm, and kyphosis improved to 37° (Figure 2A). He subsequently underwent three planned distraction procedures. After the first distraction, the curve reduced to 32°, thoracic height increased to 195 mm, and kyphosis to 35° (Figure 2B). After the second distraction, the curve reached 22°, thoracic height 210 mm, and kyphosis 30°. After the third, continued gradual improvement was noted, maintaining balanced coronal and sagittal alignment. Throughout follow-up, he remained neurologically intact, tolerated each distraction without difficulty, and exhibited progressive improvement in trunk symmetry. No wound issues, rod breakage, or implant prominence were observed. A follow-up of 12 months after index surgery, covering serial distraction, was done. 

Case report two

A ten-year-old female was evaluated for increasing spinal deformity noted during school health screening. Her parents recalled a mild curvature earlier, but a more noticeable asymmetry developed over the previous year. She had no congenital anomalies, neuromuscular conditions, or syndromic features. She remained active without pain.

**Figure 3:**
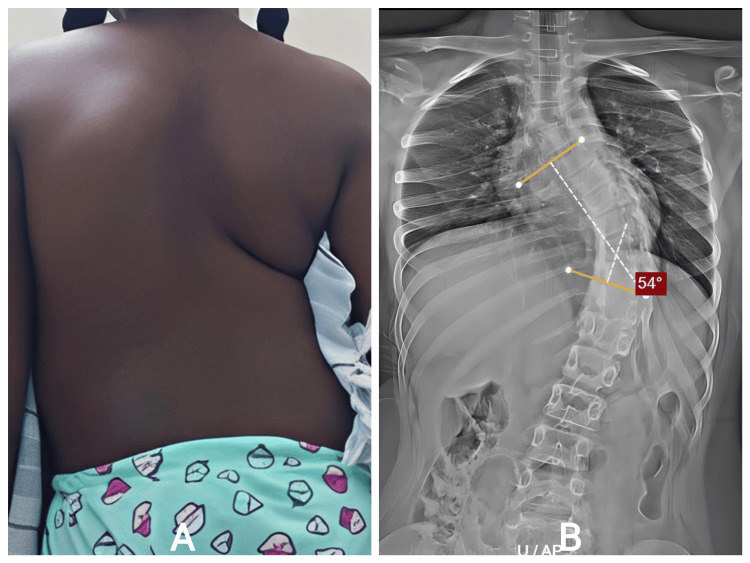
A: Standing posterior view showing thoracic asymmetry, rib prominence, and mild compensatory lumbar shift consistent with early-onset scoliosis. B: Progressive early-onset thoracic scoliosis measuring 54° (Cobb T5–T10) with a compensatory thoracolumbar curve. T5: thoracic vertebra.

Clinical examination revealed significant thoracic asymmetry, rib prominence, and mild compensatory lumbar curvature. Neurological examination was normal. Radiographs showed progressive early-onset scoliosis involving the thoracic and thoracolumbar regions. The curve magnitude and documented progression indicated that nonoperative therapy would not control deformity (Figure 3).

She underwent T3-L3 growth rod application. Intraoperative correction was achieved after careful soft-tissue release. Immediate postoperative radiographs demonstrated meaningful improvement in spinal alignment.

**Figure 4:**
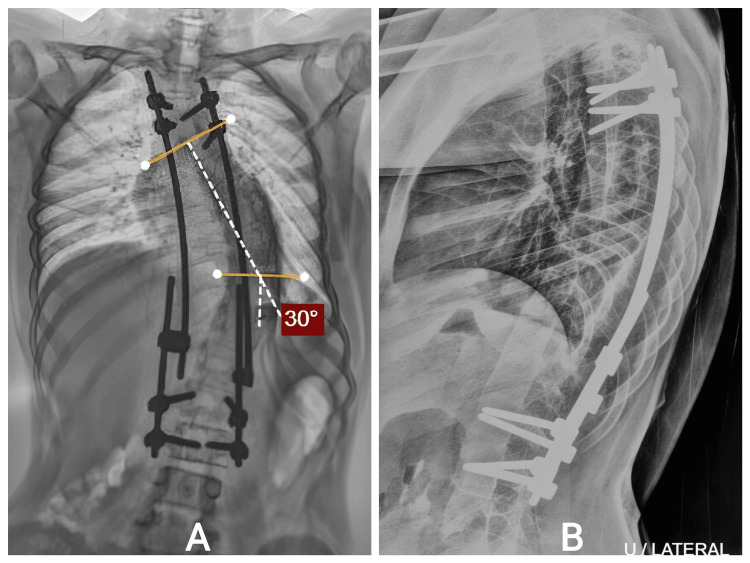
A: Progressive early-onset thoracic scoliosis measuring 54° (Cobb T5–T10) with a compensatory thoracolumbar curve. B: lateral view. T5: thoracic vertebra.

She completed multiple distraction procedures as scheduled. After each distraction, radiographs revealed progressive improvement and thoracic expansion (Figure 4). Clinically, trunk balance improved steadily. No complications were encountered: no wound breakdown, implant prominence, rod fracture, or neurologic deficits. By follow-up completion, she demonstrated excellent functional status and cosmetic satisfaction.

Case report three

**Figure 5:**
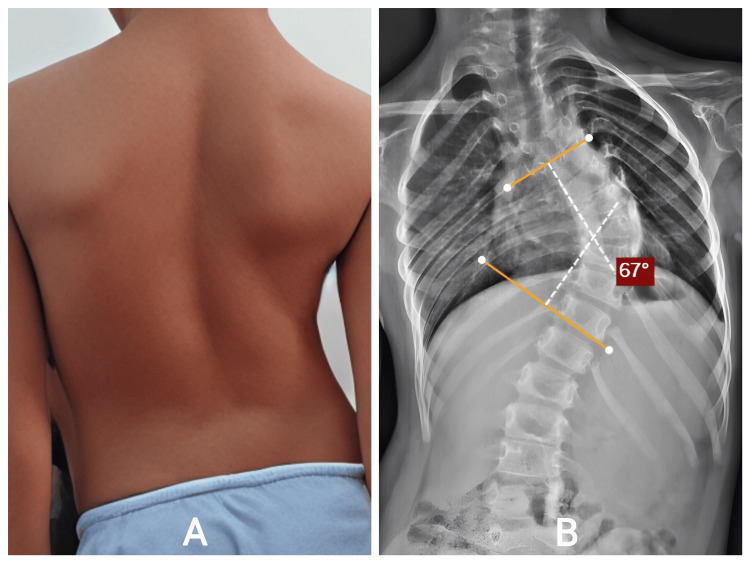
A: Posterior standing view demonstrating right thoracic asymmetry and rib prominence. B: Full-spine AP view reveals a 67° congenital thoracic scoliosis (T5–T11) with vertebral formation anomalies. AP: antero-posterior; T5: thoracic vertebra.

An eight-year-old female with congenital scoliosis involving T5-T11 presented with a visible back deformity noted since early childhood. Her parents reported that the curve appeared to worsen during growth spurts. She had no respiratory symptoms and a normal neurological examination, but had noticeable thoracic asymmetry and rib prominence (Figure 5A). Radiographs demonstrated a structural 67° thoracic congenital scoliosis, with associated vertebral formation anomalies consistent with hemivertebrae or block vertebrae (Figure 5B). Given the structural nature of the deformity and expected progression, growth rod surgery was recommended to permit thoracic development while controlling the curve.

**Figure 6:**
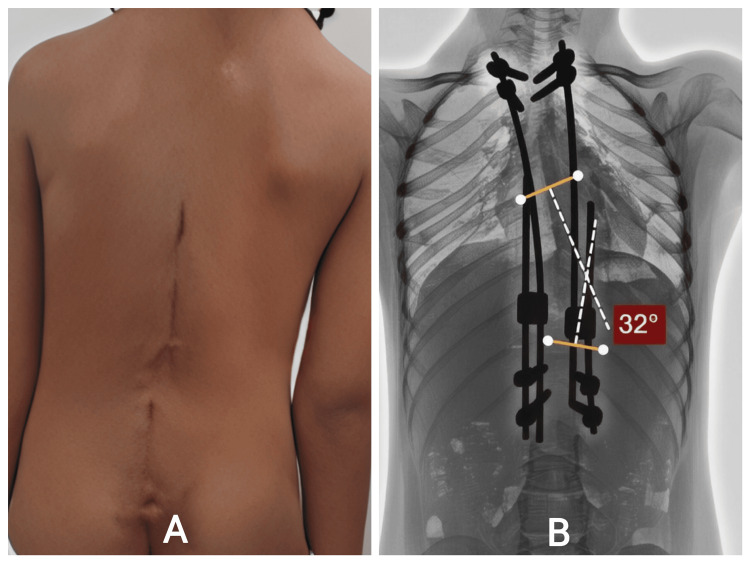
A: Follow-up Clinical Photographs – Lateral Views. B: Post–growth rod application images showing satisfactory partial correction with balanced coronal and sagittal alignment.

She underwent growth rod application across the deformity, achieving satisfactory partial correction. Postoperative alignment improved significantly. She later underwent scheduled distraction procedures, each showing incremental straightening and thoracic heightening (Figure 6). Her postoperative recovery was uneventful. She did not develop infection, hardware prominence, or mechanical failures. Clinically, she demonstrated improved posture, more symmetric thoracic contour, and preserved mobility. Her growth trajectory remained appropriate for age, and there were no neurological or respiratory concerns during follow-up.

Case report four

**Figure 7:**
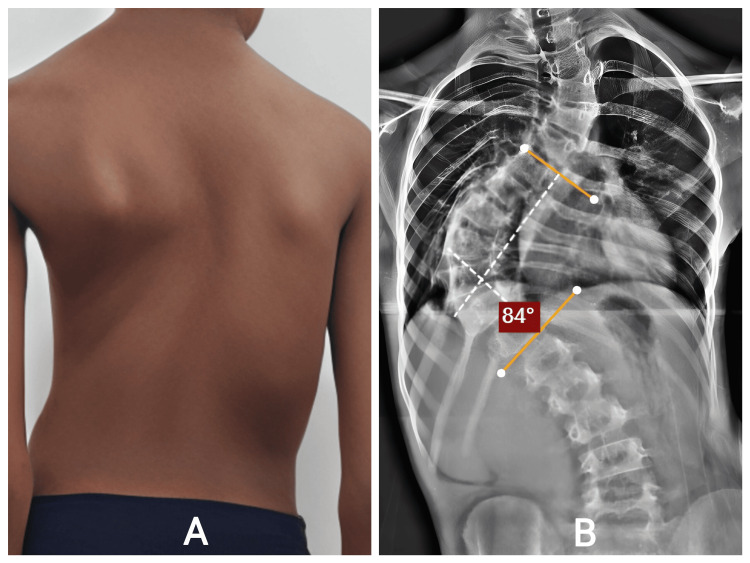
Posterior standing view demonstrating right thoracic curve, shoulder imbalance. B: AP radiograph shows right thoracic scoliosis with dystrophic features typical of NF-1. NF: neurofibromatosis; AP: anteroposterior.

The fifth patient, a child aged between eight and ten years, was evaluated for spinal asymmetry noted during routine examination. Parents observed progressive shoulder height difference and rib prominence over one year (Figure 7A). She had no syndromic associations or neuromuscular deficits. Examination confirmed a flexible thoracic curve with mild lumbar compensation; neurological examination was normal. Radiographs showed early-onset scoliosis with documented progressive curvature (Figure 7B). Observation alone was insufficient due to her young age and the curve behavior. Following detailed counselling, she underwent growth rod application designed to control deformity and allow spinal growth.

**Figure 8:**
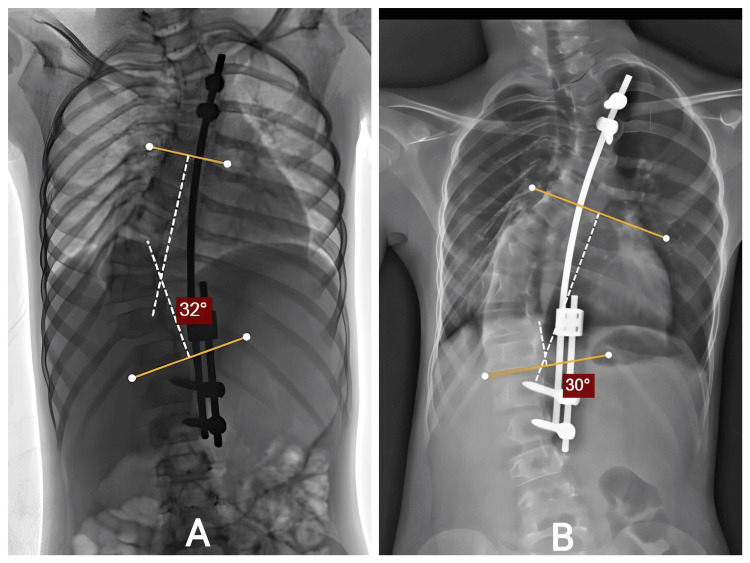
A: Posterior view demonstrating well-healed wound with improved trunk and shoulder alignment. B: Correction maintained with continued clinical improvement.

Intraoperative correction was achieved safely, and postoperative films confirmed improved alignment. She underwent serial distraction procedures, each contributing to gradual coronal and sagittal improvement (Figure 8). Clinically, her posture improved, respiratory effort remained normal, and she continued all daily activities without limitation.

No postoperative or mechanical complications occurred. She tolerated each distraction well, and no adjustments outside planned surgeries were required. The staged lengthening approach supported both cosmetic and functional outcomes.

## Discussion

Early onset scoliosis (EOS) represents a distinct and challenging group of spinal deformities due to its potential to interfere with normal thoracic and pulmonary development during early childhood. Progressive spinal curvature at this age can lead to thoracic insufficiency, reduced lung volumes, and long-term cardiopulmonary dysfunction. Historically, early posterior spinal fusion was used to halt deformity progression, but long-term studies have shown that fusion at a young age significantly compromises thoracic growth and pulmonary function, resulting in restrictive lung disease and truncated trunk height in adulthood [5]. These limitations prompted the evolution of growth-preserving techniques designed to maintain deformity control while allowing continued spinal and thoracic expansion. In this context, growth rod constructs have become an important tool in the management of EOS. The cases described in this series illustrate the benefits of growth rod distraction in children with etiologically diverse scoliosis, including congenital scoliosis and syndromic causes. Despite variability in pathology and curve rigidity, all children demonstrated progressive improvement in spinal alignment and thoracic height following staged distractions. This experience is consistent with broader evidence showing that elongated growing spine techniques can provide both coronal correction and enhanced thoracic development throughout growth [6].

Preserving thoracic volume and encouraging normal lung development remain central goals in EOS management. The thorax must grow adequately during childhood for alveolar multiplication and chest wall expansion to occur normally. Growth rod distraction promotes this process by enabling controlled spinal elongation at regular intervals. Studies evaluating elongated spinal constructs have demonstrated measurable gains in T1-T12 height with each distraction, along with improved clinical posture and respiratory mechanics [6]. In our series, radiological improvement after each distraction was evident, reinforcing the biomechanical rationale behind growth-friendly instrumentation. A notable strength demonstrated in these cases was the favourable tolerance of distraction procedures. Follow-up completion in this series occurred after completion of planned distraction cycles over a period ranging from 12 to 24 months. All children underwent scheduled lengthenings without neurological complications, wound issues, or implant failures. The thoracic height gains represent growth achieved over the documented follow-up period rather than immediate postoperative correction. Although complication rates in the literature vary, ranging from mechanical failures to soft-tissue problems, our series did not encounter such difficulties. Larger multicentre studies show complication risks rising with the number of distractions performed, particularly rod fatigue fractures and anchor pullout [7]. The absence of complications in our group may reflect meticulous surgical planning, careful distraction technique, and relatively short follow-up duration.

Growth rod lengthening has also been linked with important psychosocial outcomes. EOS can cause visible deformity, impair self-esteem, and reduce participation in physical activities. Improvement in trunk symmetry, clothing fit, and posture is often highly valued by families [8]. Previous studies examining outcomes of growing rod lengthening have highlighted improvements in body image and activity levels following deformity correction [9]. The parents of children in our series consistently reported such benefits, underscoring the qualitative impact of growth-preserving treatment. Congenital scoliosis poses unique challenges because vertebral formation anomalies contribute to curve rigidity and inevitable progression. Observation alone is rarely sufficient, and early fusion can severely limit thoracic development. Growth rod constructs offer an alternative by controlling progression while preserving growth potential. Several authors emphasize that achieving adequate thoracic length by adolescence is crucial for optimal pulmonary function [10]. In our congenital cases, staged distraction allowed continued thoracic expansion and improved alignment, demonstrating the utility of growth rods even in structurally rigid curves.

As technology evolves, newer systems such as magnetically controlled growing rods (MCGR) have emerged to reduce the morbidity associated with repeated surgeries. Early reports show encouraging results, with reduced anesthesia exposure and comparable curve correction [11]. Compared with magnetically controlled growing rods, traditional growing rods are substantially more cost-effective but require repeated surgical lengthenings under anesthesia and experienced surgical teams. Magnetically controlled growing rods reduce the need for repeated surgeries and anesthesia exposure; however, their higher cost and limited availability restrict their widespread use in resource-limited settings. Despite requiring multiple surgeries, traditional distraction-based constructs remain effective and widely applicable.

Limitations of the study

This case series has limitations inherent to its design. The sample size is small, the follow-up duration is limited, and pulmonary function assessments were not available. Additionally, the heterogeneity of etiologies prevents direct comparison between cases. Pre-treatment growth velocity data and serial pulmonary function measurements were not uniformly available for all patients and, therefore, could not be systematically analyzed in this retrospective case series. Growth rod distraction provides controlled deformity modulation rather than definitive correction, and its effectiveness may be limited in very rigid curves, severe kyphotic deformities, dystrophic scoliosis, or curves with extreme magnitudes, where only partial correction and gradual stabilization rather than complete correction can be expected. Despite these constraints, the consistent radiographic and clinical improvements observed across all children contribute meaningful real-world evidence in support of growth rod distraction.

## Conclusions

Growth rod instrumentation remains a valuable and practical strategy for managing early onset scoliosis, particularly in young children, where preservation of spinal and thoracic growth is essential. Across this case series, staged distraction provided consistent improvement in deformity correction, thoracic height, and overall clinical posture, regardless of underlying etiology. All children tolerated procedures well, and no major complications occurred during the observed follow-up period. These findings support the role of growth rods as an effective fusion-sparing alternative in resource-limited settings, helping maintain spinal balance and thoracic development during critical growth years.
